# Supplementary material for: Explaining the UK’s ‘high-risk’ approach to type 2 diabetes prevention: findings from a qualitative interview study with policy-makers in England
Source: BMJ Open. 2023 Feb 7;13(2):e066301. doi: 10.1136/bmjopen-2022-066301 (PMC9906176; doi:10.1136/bmjopen-2022-066301)
Supplement: Supplementary data [file bmjopen-2022-066301supp001.pdf]

Example Topic Guide for Public Health Policy Makers

- Stakeholder description of policy background and current role
- Description of current work portfolio
- Discussion of the role of public health in diabetes prevention (locally/nationally)
- Discussion of the public health transitioning to local authorities and challenges and opportunities associated with this.
- What funding challenges have public health faced.
- Discussion on the strategies used to engage with political leaders (locally/nationally)
- Skill sets used by public health use to exert their influence on decision making and policy development.
- Discussion on the relationship between Public Health and the NHS, particularly since the 2012 transition.
- What population-level strategies/policies are public health currently targeting. What are the challenges with taking this approach.
- How does the local/national political climate influence what is possible with regards to diabetes prevention policy.
- What kind of research evidence is used by policy makers and how is this used? Give examples.
- What are the main research-policy gaps from the policy maker's perspective.
